# Necrotic ulcerative stomatitis in a patient with long-standing celiac disease: a case report

**DOI:** 10.3325/cmj.2021.62.518

**Published:** 2021-10

**Authors:** Marijan Kovačić, Ivan Kovačić, Matea Veršić

**Affiliations:** 1 Department of Otorhinolaryngology, Zadar General Hospital, Zadar, Croatia; 2 Department of Surgery, Zadar General Hospital, Zadar, Croatia

## Abstract

Celiac disease is the most common chronic gastroenterological disease. One of the extraintestinal manifestations of this multifaceted disease are changes in the oral mucosa. However, ulceration leading to the destruction of the soft and hard tissues of the orofacial region has not been reported so far. We report on the development of necrotizing ulcerative stomatitis in a 41-year-old woman with celiac disease. The initial ulcerative lesion was located in the lower lip mucosa. Necrosis of all layers of the left side of the lip and oral commissure progressed very quickly. The resulting defect required plastic reconstructive surgery. We successfully compensated for the defect by applying a combination of two flaps from the remaining tissue of the lower lip. Oral competence was established immediately after the operation, and a very good esthetic appearance two months later.

The epidemiological link between celiac disease and oral mucosal ulcers is well known (1). Although this disease may cause malabsorption as a major factor in the development of necrotizing ulcerative stomatitis (NUS), such a case has not been reported in the literature. The onset of this infection is preceded by poor dental and gingival status accompanied by gingival ulcers and necrotic periodontitis. In patients with impaired immune system and malnutrition oral infection develops very rapidly into NUS. At this stage, the patient can develop septicemia and fatal outcome or the inflammation is defined by a well-demarcated circular defect called noma (2). NUS usually occurs in children living in poor conditions in Central African countries. In the developed world today, it is encountered sporadically, most often in seropositive HIV patients (2,3).

## Case presentation

**Figure 1:**
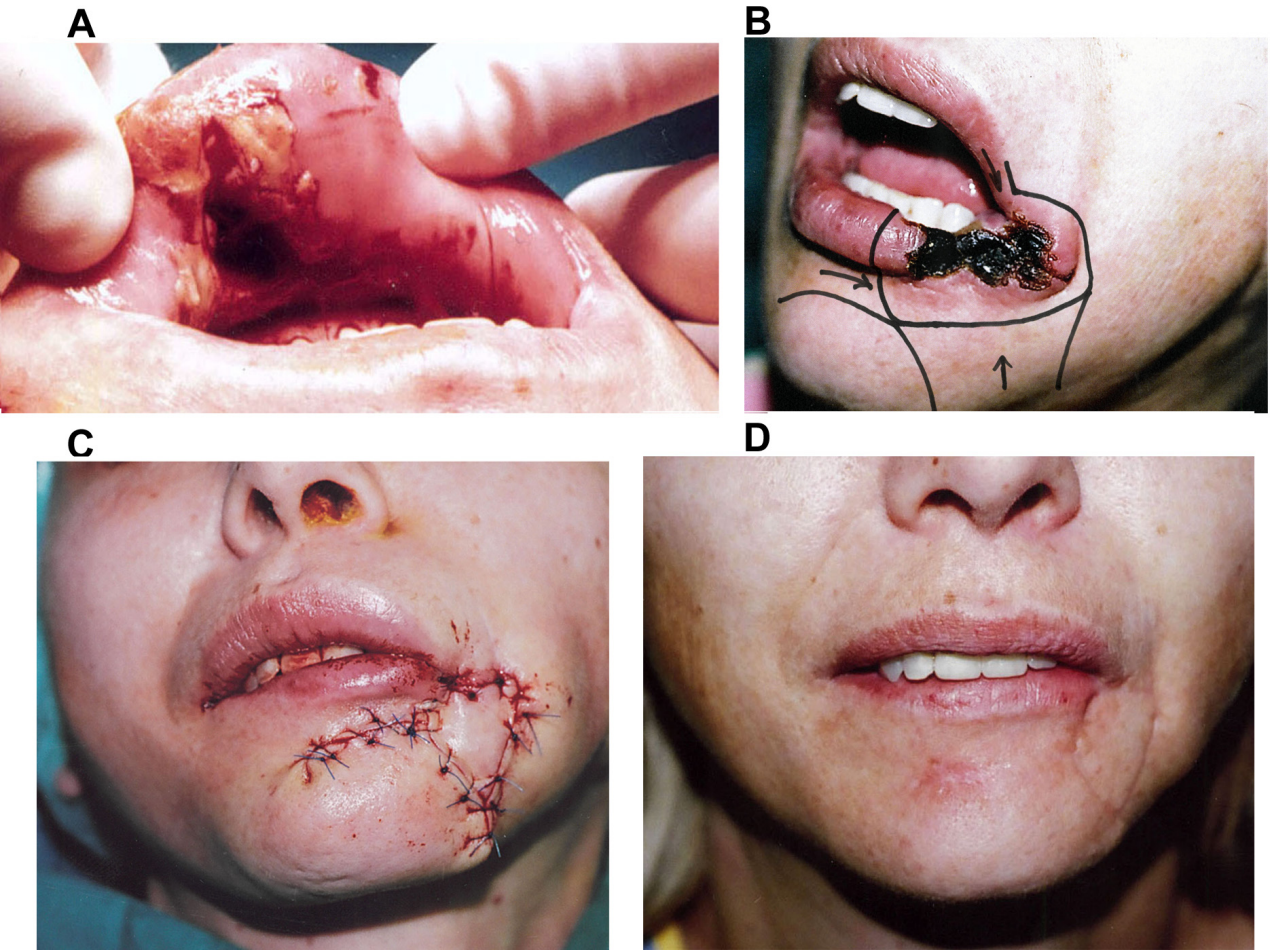
(**A**) Ulcer-necrotic changes of the mucosa and muscles of the lower lip. (**B**) A segment of the lower lip and oral commissure affected by necrotizing inflammation, and a reconstruction plan for the formation of two advancement flaps. (**C**) Immediate postoperative view. (**D**) The result of reconstruction after three months. The lip is symmetrical with a natural color and texture of the vermilion.

A 41-year-old woman (body mass index 18.2) was admitted to our hospital in poor general condition. She was highly febrile and dehydrated, with a swelling of the soft left side of her face. In the oral cavity in the region of her lower lip, she had a deep and large ulcer (Figure 1).

A long medical history of celiac disease was recorded, with occasional oral manifestations of recurrent aphthous stomatitis. As a child, the patient contracted chickenpox and measles. In the months before admission she felt muscle weakness and difficulty moving. She was being treated for an anxiety-depressive disorder. Regarding medications, she was taking only Xanax. Her appetite was poor and she was consuming an unbalanced diet. In the last two-three months, she experienced bloating with alternating constipation and profuse diarrhea. The patient denied acute and chronic respiratory diseases, skin, genital and perianal changes, orogenital contact and sexual activity, eye problems, photosensitivity, and arthralgias. She did not experience mechanical, chemical, and thermal trauma to the oral cavity before the ulcer appeared, nor did she injure herself. Her menstrual cycles were irregular for some time, and urination was without diuresis.

Routine laboratory tests showed an elevated leukocyte count of 19.2/mm^3^ with an appearance of less differentiated neutrophils (19%), high C-reactive protein (174 mg/L) and erythrocyte sedimentation rate (61/mm/h), erythrocytes 3.37 × 10^12^, hemoglobin 89 g/L, hematocrit 0.34 L/L, and blood glucose level 3.5 mmol/L. After rehydration, we started antimicrobial intravenous amoxicillin therapy with clavulanic acid and metronidazole, and began gradual nutritional rehabilitation. Chlorhexidine was used for oral cavity care. Additional biochemical analysis yielded lower values of total protein (61 g/L), albumin (32 g/L), iron (2.8 μmol/L), ferritin (11 μg/L), calcium (1.92 mmol/L), copper (11.2 μmol/L), 25-OH D (53 nmol/L), vitamin B12 (102 nmol/L), and folic acid (7.6 nmol/L). Tumor markers and thyroid hormone findings were within the reference range (CA 125; CA; CEA; TSH; fT4; fT3, HtgAt <20.0 IJ/mL). The tests for HIV infection and antineutrophil cytoplasmic antibodies, antinuclear antibody, anti-nuclear factor, herpes simplex virus type 1 + 2, herpes zoster virus, Waaler Rose test, hepatitis B surface antigen, and hepatitis C antibody were negative, while tissue transglutaminase antibodies were >10. The bacterial culture swab was negative and *Streptococcus intermedius* was isolated in the tissue culture, sensitive to the applied therapy.

**Figure 2:**
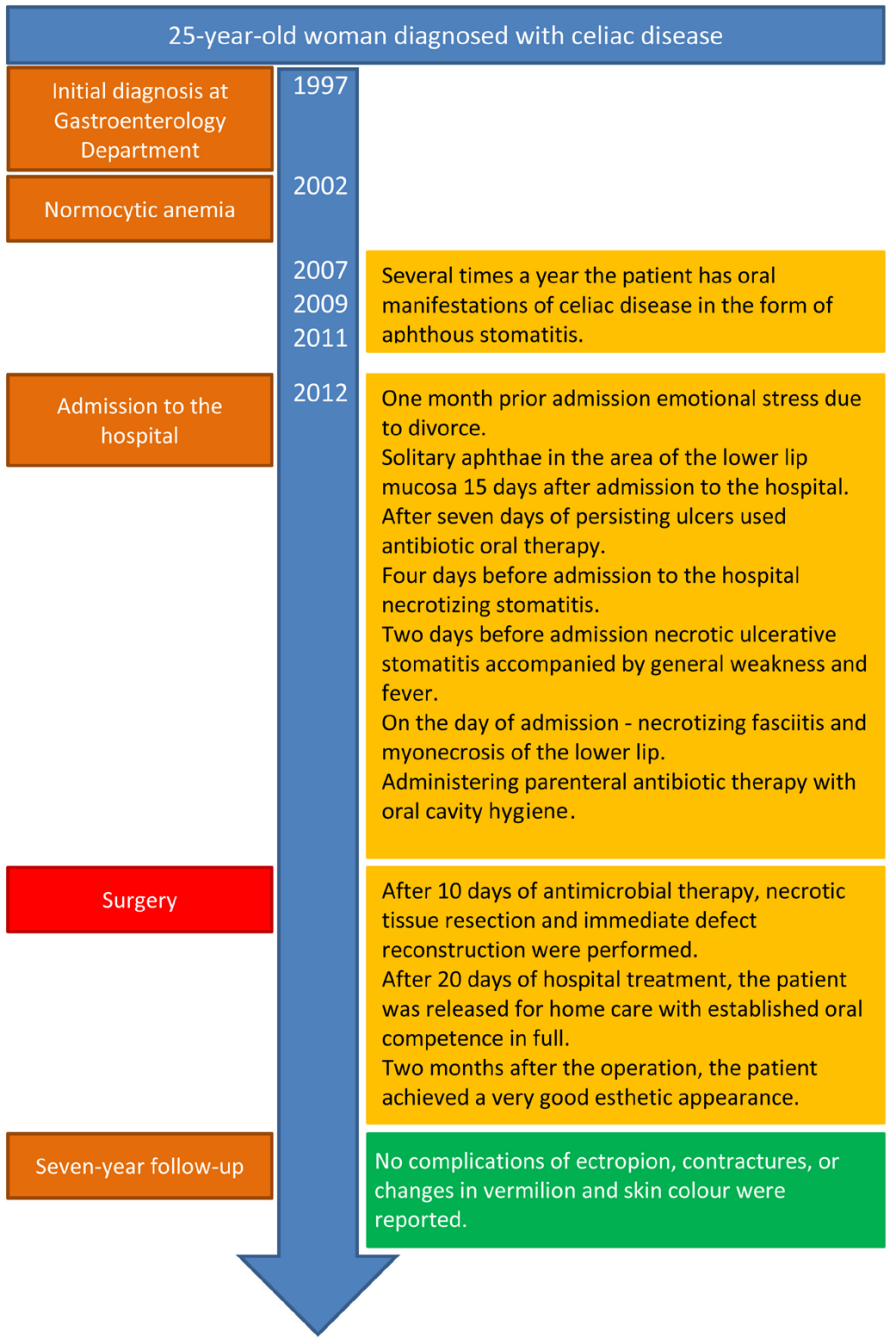
The timeline of diagnostic and clinical procedures.

A week after conservative treatment, the patient's general condition improved significantly. A few days later, we performed extensive debridement and immediate reconstruction of the resulting defect. We used two local advancement flaps formed from the tissue of the remaining part of the lip and the tissue belonging to the mental region (4,5). In addition to acute inflammatory cells, histopathological findings showed extensive tissue necrosis in all layers and vascular thrombosis, without malignancy and formed granulomas. A high-quality esthetic appearance of the oral commissure and the symmetry of the lower lip were achieved after two months, without any functional problems. The patient was followed-up for seven years, during which period there were no recurrences of necrotic inflammation in the oral cavity or other oral celiac disease manifestations (Figure 2).

## Discussion and conclusions

Celiac disease can be successfully treated by strictly respecting a gluten-free diet. Any non-adherence to such a diet pattern changes the intestinal flora and oral microbiota, resulting in the abdominal and oral manifestation of the disease (1,6). The occurrence of small multiple or single larger ulcers in the oral cavity in this disease is not uncommon. Campisi et al (7) reported a significantly higher presence of aphthous stomatitis in patients with celiac disease than in healthy individuals (22.7% vs 7.1%). Our patient experienced several episodes of aphthous stomatitis (recurrent aphthous stomatitis), but just before the onset of NUS, she developed solitary macro aphthae. The lesion did not remain at the level of the mucosa and lamina propria but penetrated into the deeper layers of the lower lip and seriously endangered the patient's health. Because ulcers in the oral cavity can be an initial manifestation of a number of other diseases, we conducted an additional clinical and laboratory examination. We excluded the possibility of trauma lesions, self-harm, drug use, autoimmune diseases, dermatosis, systemic infections, and other inflammatory conditions. The histological analysis of the removed part of the lower lip excluded malignant disease, granulomatous inflammation, necrotizing sialometaplasia, and other diseases of the small salivary glands.

The occurrence of NUS in the developed countries is rare and usually takes place in HIV-positive patients, and in several cases has been reported as the initial disease of an undiagnosed HIV infection (3). In patients with Crohn’s disease, only one case has been described so far (8). Besides the primary polymicrobial infection of the oral lesion, other co-factors are required for the development of this destructive progressive gangrenous inflammation. Their interaction is very complex and still difficult to understand (2). In our patient, the disease was probably triggered by an unbalanced and poor-quality diet with a reduced intake of proteins, albumin, vitamins, and electrolytes. Stress further exacerbated the gastrointestinal symptoms and consequently caused intestinal dysbiosis, which disrupted adequate response of the immune system (6). Changing oral microbiota and contamination of the initial lesion by microorganisms from the immediate environment, and the synergistic action of these processes with the inflammatory process caused NUS (9).

The local and general status of our patient indicated the severity of the situation and the need for hospital treatment. By administering broad-spectrum intravenous antibiotics, fluids, electrolytes, and various dietary supplements, we stopped the progression of inflammation and prevented a greater destruction of the soft and bone tissue of the face. We did not change the antibiotic therapy due to a good clinical response and not due to the susceptibility of the isolated pathogen (*Streptoccocus intermedius*). This inflammatory process was caused by a heterogeneous group of microorganisms, and the validity of the results of microbiological analyses is questionable. Different microorganisms may be present at different stages of this disease, and their isolation has no certain etiological significance (9).

After the acute disease phase, a larger or smaller defect of soft and bone tissue always remains. There is no standard surgical approach to treating the effects of NUS. A common practice is to delay the reconstruction for several months to a year in order to prevent a secondary infection of the defect (10). We opted for an early intervention for two reasons – the request of the patient and the absence of osteonecrosis. After an extensive excision of the inflamed soft tissue of the lower lip, we successfully compensated for the defect by applying a combination of two flaps. Oral competence was established immediately after the procedure, and the final very good esthetic result two months later. The absence of contractures and atrophy of the flaps and discoloration of the vermilion and skin confirm the correctness of the chosen reconstruction method. A responsible and dedicated approach to the patient's diet and mental stability prevented the recurrence of ulcers in the oral cavity during the seven years of follow-up.
